# Minimal surgery for tractional retinal detachment secondary to branch retinal vein occlusion: a case report

**DOI:** 10.1186/s13256-022-03496-3

**Published:** 2022-07-23

**Authors:** Alper Bilgic, Aditya Sudhalkar

**Affiliations:** 1 Alphavision Augenarzt Practice, Buergermeister-Smidt Str. 162, 27568 Bremerhaven, Germany; 2 Sudhalkar Eye Hospital and Retina Centre, Baroda, India

**Keywords:** Tractional retinal detachment, Retinal vein occlusion, Endo-tamponade, Visual outcomes, Minimal surgery

## Abstract

**Background:**

Tractional retinal detachment secondary to retinal vein occlusion is a complex entity that can be extremely difficult to manage due to an intricate association of the retinal tissue with the fibrovascular proliferation, making vitreous dissection an extraordinarily difficult procedure. Minimal surgery without endo-tamponade can reduce recovery time and avoid complications of surgery, which in some cases can lead to blindness and even phthisis.

**Case presentation:**

A 64-year-old Indian woman presented with progressive worsening of vision (right eye) due to fovea involving tractional retinal detachment secondary to supero-temporal branch retinal vein occlusion. After anterior, core and peripheral vitrectomy, the epicenter of the fibrous bridge causing foveal split was identified and released. The corrected distance visual acuity improved from 6/60 pre-operatively to 6/12 post-operatively. At the 5-year follow-up, the patient remains stable both anatomically and visually.

**Conclusions:**

This case illustrates how careful identification of the epicenter of traction helps maximize visual gain in patients with minimal risk of iatrogenic retinal tears and eliminates the need for endo-tamponade with either gas or silicone oil. Minimal surgery for tractional detachment provides excellent visual gains with minimal risks in select cases.

## Background

Tractional retinal detachments secondary to retinal vein occlusion are an important cause of significant visual loss and require surgical management when the macula is involved or when there is extensive neovascularization and an impending vitreous bleed and/or chance of rhegmatogenous breaks [1, 2]. Pars plana vitrectomy with endo-tamponade is the treatment of choice, but the procedure is rendered difficult by extensive fibrovascular proliferation closely associated with and attached to the retina [3–5]. The situation is complicated by the presence in most cases of macular ischemia and generalized compromise of retinal function [5, 6]. Visual gains in such cases are extremely varied (between 20/40 and 20/400) [6], and largely poor given the ischemic nature of the disease and its complications, regardless of endo-tamponade use. Indeed, extensive surgery almost always necessitates the use of endo-tamponade.

The surgery itself can be complicated by iatrogenic retinal breaks and eventual total retinal detachment, leading to permanent loss of vision and even phthisis [5–7]. Here, we described a unique and interesting interesting case of how tractional retinal detachment with macular involvement secondary to retinal vein occlusion can be treated with minimal surgery, thereby avoiding the use of tamponades and subsequent complications, as well as ensuring early visual recovery.

## Case presentation

**Fig. 1:**
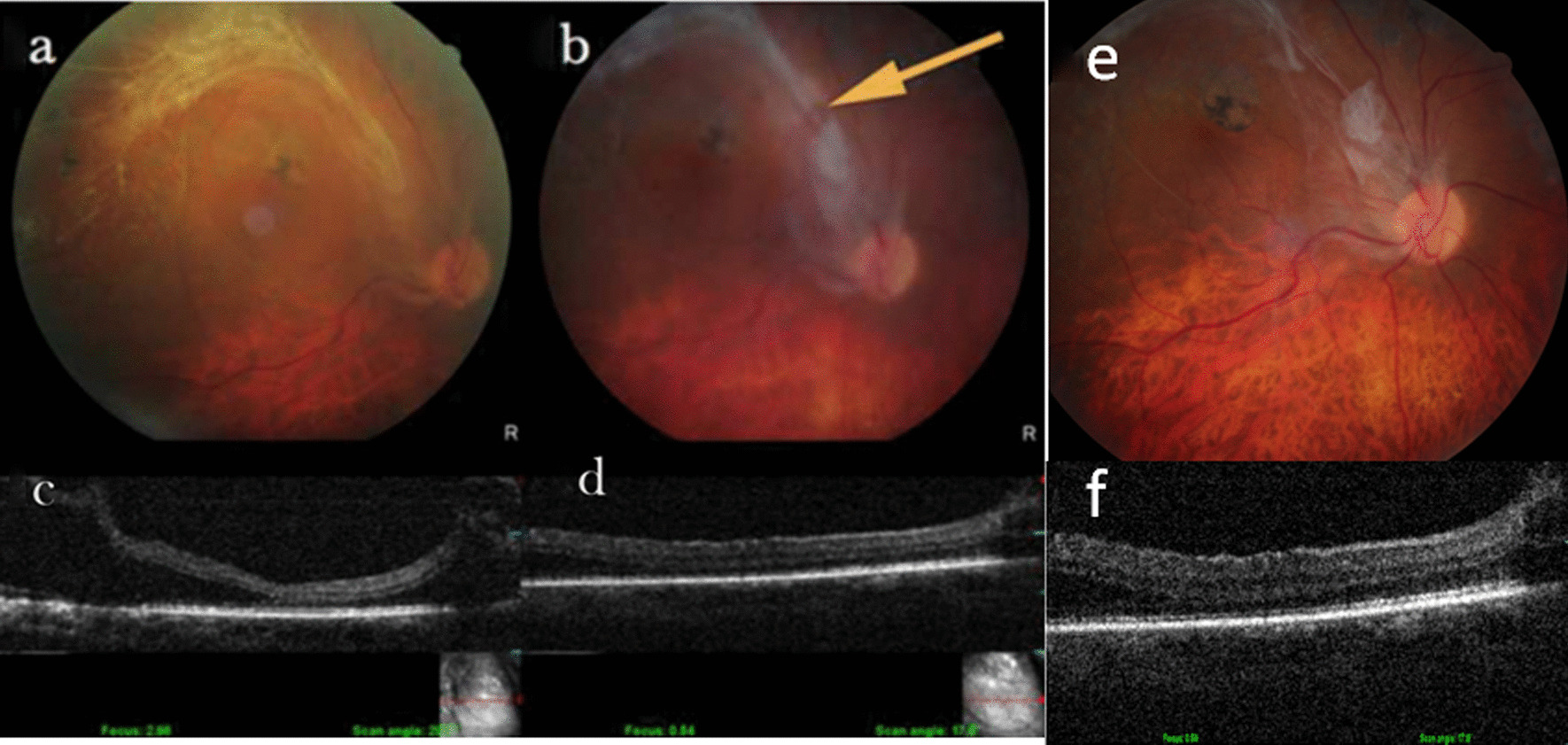
Preoperative state: the tractional retinal detachment seen in **a** (clinical photograph) and **c** (OCT scan). This was largely relieved with transection of the epicenter of the fibrous band along the supero-temporal arcade **(b**; clinical photograph, yellow arrow) and **d** (OCT scan) after vitrectomy. The relief in traction has been stable over a 5-year follow-up, as evidenced by the fundus photograph (**e**) and OCT scan (**f**). The arrow points to cleavage in the fibrous band which helped settle the retina. *OCT* Optical coherence tomography

**Fig. 2:**
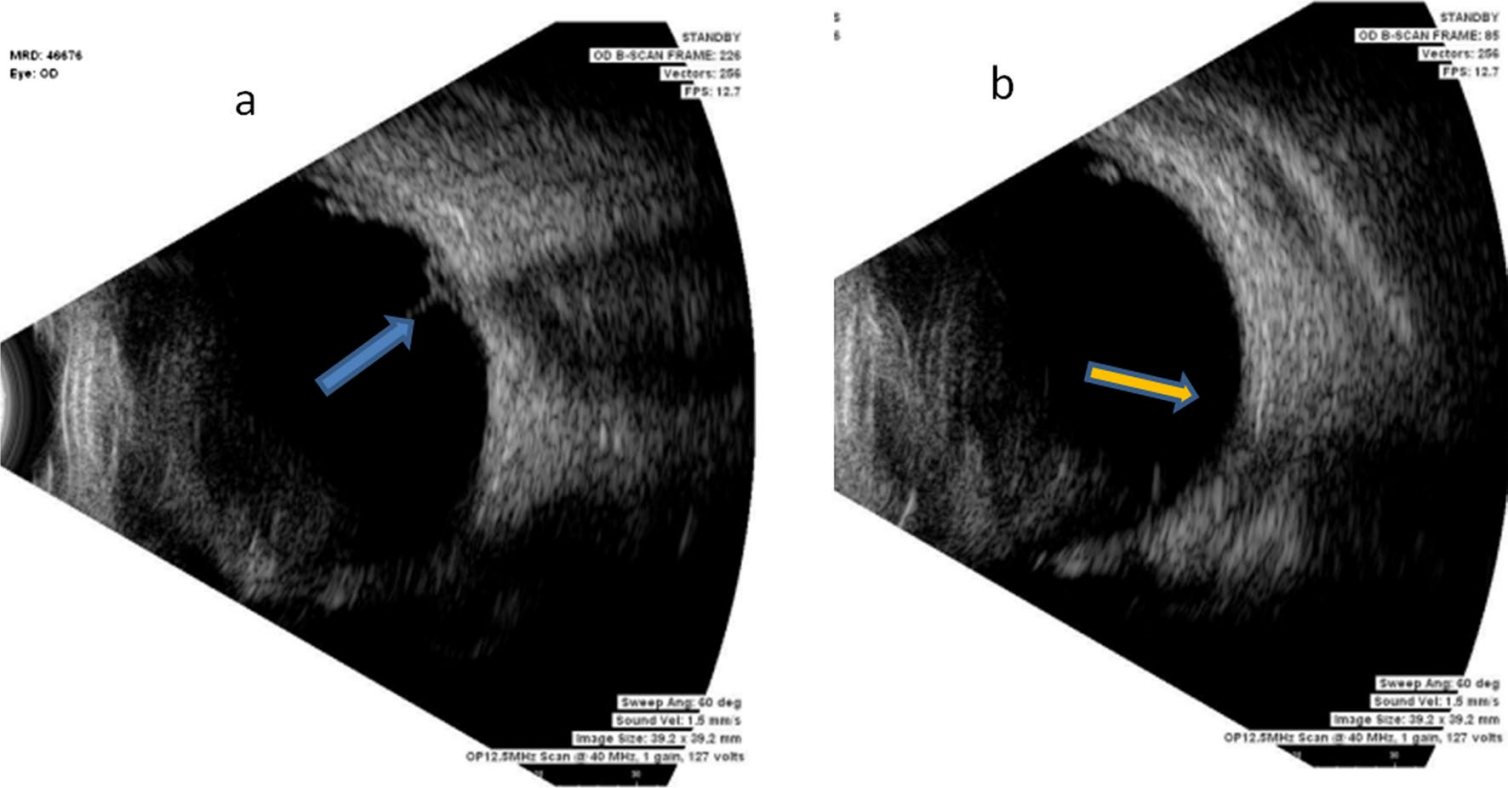
Preoperative ultrasound scans (**a**) of the patient demonstrates obvious traction adjacent to the disc (blue arrow). Ultrasound B scan at the 5-year follow-up shows that the traction remains relieved in the area adjacent to the disc (**b**). The patient’s vision recovered to 6/12 and has been maintained over 5 years. In the Ultrasound, the blue arrow points to the tractional detachment preoperatively and the yellow arrow points to the released traction postoperatively

A 64-year-old Indian woman presented with progressive worsening of vision (right eye) due to fovea involving tractional retinal detachment secondary to superotemporal branch retinal vein occlusion. Her vision at presentation was 6/60 (logMAR 1.0) in the right eye and 6/6 (0.0 logMAR) in the left eye. Anterior segment examination revealed pseudophakia in both eyes. The intraocular pressure was 12 mm Hg in the right eye and 16 mm Hg in the left eye. She was diagnosed to have tractional retinal detachment (Fig. 1a) involving the macula in the right eye. The results of the left eye fundus examination were normal. She had a 10-year history of hypertension that was well-controlled on medication; otherwise, she had no clinically relevant family/social history. Given that the macula was involved in the tractional detachment, a decision was made to perform pars plana vitrectomy with membrane peeling and endolaser therapy with or without silicone oil (depending upon the finding of rhegmatogenous breaks, either pre-existing or iatrogenic, during surgery). The surgery was performed using the 23-gauge Constellation Vitrectomy System (Alcon, Fort Worth, TX, USA). This gauge was chosen as there was a lack of stock of 25 G and 27 G sets in India at the time of surgery. Infusion was initiated at 7:00 a.m. through valved micro-cannulae. After anterior, core and peripheral vitrectomy, the epicenter of the fibrous bridge causing foveal split was identified and released using the vitreous cutter (Fig. 1b). The retina was observed by fall in place following this action. Further procedures could be avoided through proper identification of the epicenter of traction, and the use of intraocular gas/silicone oil was avoided. The eye was left with aqueous tamponade and examined the next day. The optical coherence tomography image showed that the retina had fallen back in place and it continued to re-attach over the following week. At 7 days postoperative, vision had improved to 6/12 (0.3 logMAR), and vision has beenen maintained over 5 years of follow-up. The postoperative B scan (Fig. 2) at the 5-year follow-up shows stable traction

## Discussion and Conclusion

Tractional detachments associated with branch retinal vein occlusions are often considered to be among the most difficult to operate on [1–4], and minimal intervention to reattach the fovea should be the mainstay of treatment [5–7]. The primary challenge lies in the identification of a plane between the posterior hyaloid (so-called ‘second membrane’) and the retina [1, 4, 7, 8]. Non-identification of the membrane can lead to retinal breaks and convert a tractional retinal detachment into a combined tractional and rhegmatogenous retinal detachment, prompting a more thorough and more arduous clean-up of traction all over. This can be particularly difficult in an ischemic retina that tears readily at the slightest touch. The current general consensus in retinal surgery is one of minimum intervention, a change in strategy from the earlier adopted process of comprehensive removal of traction. The case presented here is an example of the latter approach. This case illustrates how careful identification of the epicenter of traction helps maximize visual gain in patients with minimal risk of iatrogenic retinal tears and eliminates the need for endo-tamponade with either gas or silicone oil. Endo-tamponades come with their own set of problems, such as lack of early visual rehabilitation, glaucoma, corneal decompensation and cataract formation. Complications for tractional detachment surgery has an incidence ranging from 3 o 20% [9, 10].

 At the time of writing, the patient has been followed up for 5 years and, as seen in Fig. 1b, is stable both anatomically and visually. Past literature on minimalistic approaches for epiretinal membranes has been encouraging [11]. Reibaldi and associates reported similar visual acuity gains and improvements in retinal thickness in patients undergoing minimal surgery for retinal membranes when compared with patients who received standard vitrectomy. Indeed, the rates of nuclear cataract were significantly lower with the minimal approach, thereby improving patient quality of life. This is a solitary case report on a condition along the same spectrum but with a higher degree of traction, and further literature and similar case studies will buttress the perspective of minimal surgery.

In conclusion, minimal surgery for tractional retinal detachment secondary to retinal vein occlusion might be a good option in select cases wherein the primary problem is macular detachment in an otherwise stable eye.

## Data Availability

All supporting data is included in the manuscript.
